# Volumineux corps étranger colorectal introduit volontairement: à propos d’un cas

**DOI:** 10.11604/pamj.2019.34.142.20793

**Published:** 2019-11-13

**Authors:** Saad Slaiki, Hicham EL Bouhaddouti, Ouadii Mouaqit, Abdelmalek Ousadden, Khalid Ait Taleb, El Bachir Benjelloun

**Affiliations:** 1 Service de Chirurgie Viscérale, CHU Hassan II, Fes, Maroc

**Keywords:** Foreign body, rectal, voluntary, Kidney transplantation, left sided, left inferior vena cava

## Abstract

L'introduction de corps étrangers (CE) est une curiosité et un tabou dans notre pays. Elle se caractérise par la gravité des complications éventuelles et les différentes possibilités thérapeutiques. Nous rapportons le cas d'un patient, ayant eu une incarcération d'un énorme objet introduit volontairement par voie anale. Il a bénéficié d'une extraction manuelle. Cette dernière permet, quand elle est possible, d'éviter la chirurgie qui s'impose en cas d'échec ou de complications.

## Abstract (en)

Insertion of foreign bodies is a curiosity and a taboo in our country. It is characterized by the severity of possible complications and the different therapeutic opportunities. We report the case of a patient with rectal incarceration of a vouminous object introduced voluntarily. He underwent manual extraction. Extraction, when possible, allow to avoid surgery that is necessary in the case of failure or complications.

## Introduction

L'introduction de corps étrangers (CE) par l'anus est un phénomène bien décrit, et n'est plus considéré comme rare, en occident [1,2]. Par contre au Maroc, cela reste une curiosité et un tabou. Des objets, parfois insolite, peuvent être introduits dans le rectum à des fins thérapeutiques, sexuelles (érotisme anal ou agressions sexuelles), par trouble du comportement, pour dissimuler l'objet (drogues, armes) ou plus rarement, lors de circonstances accidentelles.

## Patient et observation

**Figure 1:**
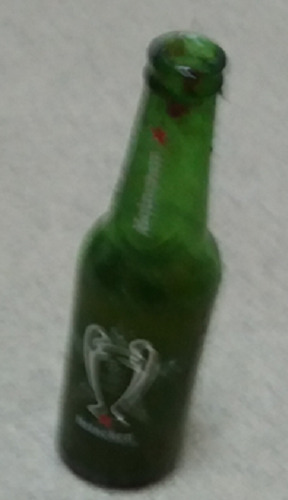
Le corps étranger après son extraction

**Figure 2:**
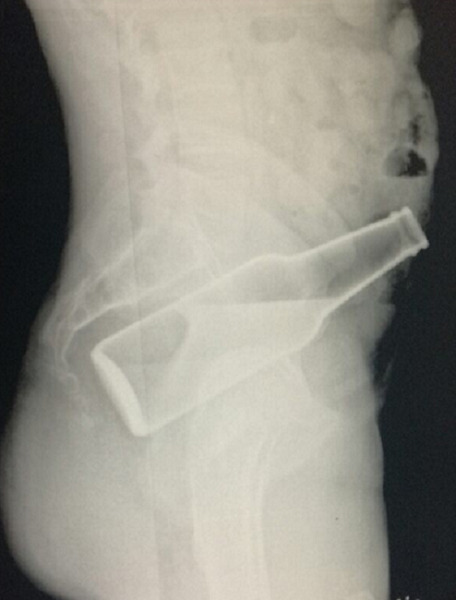
Abdomen sans préparation montrant le corps étranger au niveau du pelvis

**Figure 3:**
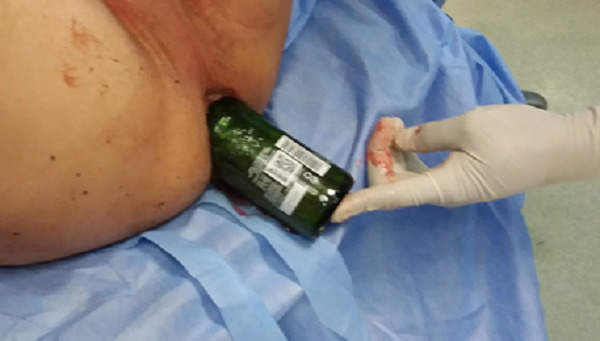
Le corps étranger lors de l´extraction

Un patient de 45 ans sans antécédents notables, s'est présenté aux urgences pour CE (Figure 1) incarcéré en intra rectal depuis son introduction un jour auparavant « au cours d'un acte sexuel ou le patient était ivre ». L'examen a trouvé un patient en bon état général avec un bout de la bouteille palpable en sous ombilical. Le toucher rectal percevait l'objet (bouteille de bière) au bout du doigt. La radiographie avait mis en évidence le corps étranger (Figure 2). L'extraction a été réalisée par voie basse sous sédation, au bloc opératoire, en position de taille périnéale. La manœuvre d'extraction ayant consisté en une petite pression sur l'abdomen au niveau du bout palpable ce qui a permis l'extraction du CE par voie basse (Figure 3). Le patient était gardé en observation après l'extraction. L'évolution était sans particularités. Le patient était déclaré sortant J + 2, puis il fut adressé en consultation psychiatrique.

## Discussion

Devant l'insertion rectale de CE, il est impératif de ne pas humilier le patient. Il faut le traiter avec le même respect montré aux autres patients. En plus d'être éthique, cela facilite la prise en charge. Le rapport le plus ancien sur la prise en charge d'un corps étranger intra-rectal remonte au XVIème siècle [3]. On distingue l'incarcération de corps étrangers ingérés par voie buccale et les corps étrangers introduits par voie rectale pour diverses raisons. La cause la plus fréquente d´insertion de corps étranger est liée aux pratiques sexuelles, la plupart du temps solitaires. Les autres étiologies sont l'auto-thérapie (d'une constipation, d'hémorroïdes, ou de prurit anal), l'origine traumatique, les agressions, et l'origine psychiatrique [4]. Notre patient rapporte l'introduction du corps étranger secondaire à un état d'ivresse lors de pratique de sexe. La présence de corps étranger intra-rectaux est très peu courante dans les pays en développement, et plus fréquente dans les pays industrialisés [5]. Les patients se présentent souvent aux urgences plusieurs heures ou plusieurs jours après l'insertion du corps étranger, avec un délai moyen de 1,9 jour [3]. Notre patient s'est présenté 1 jour après l'introduction du corps étranger. Les principaux motifs de consultation sont la rectorragie et la douleur abdominale aigüe ou persistante associée à un syndrome occlusif ou sub-occlusif. Les ténesmes ou les inconforts ano-rectaux sont souvent citées [1].

Un toucher rectal (mieux réalisé sous sédation consciente), vérifie l'intégrité anorectale et peut retrouver le CE [6]. Combiné à la palpation abdominale, il permet parfois d'estimer sa position [1]. Comme le cas de notre patient, le toucher rectal ainsi que la palpation abdominale a permis de faire le diagnostic. Si l'objet est radio-opaque, le diagnostic est confirmé à l'ASP (Abdomen sans préparation) qui visualise sa forme, sa taille et sa position, comme le cas pour notre patient. Les végétaux et objets en plastique peuvent rester invisibles ou être devinés grâce à leur silhouette. L'ASP peut aussi objectiver un pneumopéritoine, signe d'une perforation digestive, imposant la laparotomie en urgence. Une rectosigmoïdoscopie peut être tentée en faisant attention à ne pas éloigner le CE [1]. La prise en charge adéquate, implique l'extraction sans risques de l'objet avec diagnostic de toute lésion colorectale associée, qui peut être mortelle si non détectée. Le CE peut être amené, souvent sous sédation consciente, à descendre par une manipulation douce associée à une pression pelvienne, en vue d'une extraction trans-anale. Cependant, la concavité sacrée et le spasme anal tendent à retenir le CE loin de l'anus [1,7,8].

Des succès d'extraction par voie basse ont été rapportés mais concernent surtout les CE de petite taille [1]. Chez notre patient, malgré la taille considérable de l'objet, l'extraction par voie basse était réussie. Certains facteurs comme la taille, la forme et la migration des corps étrangers peuvent rendre difficiles la recherche et l'extraction par voie basse. En cas d'échec, une laparotomie s'avère nécessaire [7,9]. La laparoscopie offre des avantages potentiels, mais a été peu décrite dans la prise en charge de ce problème [11]. La laparotomie est réalisée dans moins de 40% des cas [2,10]. La colotomie peut être évitée, comme dans notre cas, surtout si la CE n'est pas large. La mise en place d'une stomie d'amont dépend du degré du traumatisme périnéal, de la chronicité de la situation, et de l'état de la paroi colorectale apprécié en peropératoire. Enfin, le support psychologique est nécessaire dans tous les cas pouvant aller jusqu'au suivi psychiatrique [11].

## Conclusion

L'introduction volontaire d'une CE reste une pathologie taboue dans notre pays. L'extraction sans chirurgie sous sédation reste le moyen idéal toutefois, le recours à la chirurgie s'impose en cas d'échec.

## Conflits d’intérêts

Les auteurs ne déclarent aucun conflit d'intérêts.
